# Nachhaltige Vergütung der B‑Zentren für Seltene Erkrankungen in Deutschland – Status quo und Lösungsansätze

**DOI:** 10.1007/s00103-022-03562-7

**Published:** 2022-07-21

**Authors:** Svenja Litzkendorf, Daniela Eidt-Koch, Jan Zeidler, Johann-Matthias Graf von der Schulenburg

**Affiliations:** 1 Center for Health Economics Research Hannover (CHERH), Leibniz Universität Hannover, Otto-Brenner-Str. 7, 30159 Hannover, Deutschland; 2 Fakultät Gesundheitswesen, Ostfalia Hochschule für angewandte Wissenschaften, Wolfsburg, Deutschland

**Keywords:** Finanzierung, Pauschalierte Vergütung, Fokusgruppen, Qualitative Inhaltsanalyse, Ambulante Versorgung, Financing, Flat fee, Focus group interviews, Qualitative content analysis, Ambulatory healthcare

## Abstract

**Hintergrund:**

Um eine spezialisierte Versorgung von Menschen mit seltenen Erkrankungen (sE) zu gewährleisten, wurden zahlreiche Zentren für Seltene Erkrankungen (ZSE) gegründet. Für die ambulante Behandlung von Betroffenen in Krankenhäusern steht dabei eine Vielzahl von Versorgungs- und Vergütungsformen zur Verfügung. Studien zu einzelnen sE ergaben bereits Hinweise auf Defizite in Bezug auf eine kostendeckende Vergütung der ZSE.

**Ziel der Arbeit:**

Untersuchung der aktuellen Versorgungs- und Vergütungsstrukturen in den ZSE und die Entwicklung von Ansätzen für zukünftige nachhaltige Vergütungsstrukturen.

**Material und Methoden:**

Mittels Fragebogenerhebung wurden zunächst ZSE in Deutschland zu ihrer Versorgungs- und Vergütungsform befragt. Im Rahmen zweier Fokusgruppen- und eines Experteninterviews mit Vertreter:innen der ZSE, der Kostenträger, der Gesundheitspolitik sowie Patient:innen wurden im Anschluss die aktuellen Versorgungs- und Vergütungsformen, Möglichkeiten der zukünftigen Gestaltung der Versorgung von Menschen mit sE sowie Ansätze für eine leistungsorientierte Vergütung diskutiert. Das Material wurde inhaltsanalytisch nach Kuckartz ausgewertet.

**Ergebnisse und Diskussion:**

39 Zentren beteiligten sich an der Fragebogenerhebung. 38 % dieser Zentren werden über eine Pauschale für Hochschulambulanzen (HSA) vergütet, deren Höhe stark variiert. 41 % weisen eine Mischvergütung aus HSA-Pauschale und weiteren Vergütungsformen auf. In den Interviews wurde eine Unterdeckung der Kosten in den ZSE mit Auswirkungen auf die Patientenversorgung benannt und zur Sicherstellung einer nachhaltigen Versorgung Handlungsbedarf zur Weiterentwicklung der Vergütungsstrukturen festgestellt. Eine „Sonderpauschale für sE“, die den besonderen zeitlichen Bedarf in der Versorgung von Menschen mit sE abbildet, wurde als möglicher nachhaltiger Vergütungsansatz präferiert.

**Zusatzmaterial online:**

Zusätzliche Informationen sind in der Online-Version dieses Artikels (10.1007/s00103-022-03562-7) enthalten.

## Abstract (en)

**Background:**

To ensure specialized care of patients with rare diseases, numerous centres for rare diseases were funded over the past few years. The reimbursement of patients’ ambulatory care in hospitals, however, is characterized by a plurality of forms of care and payment. There is some evidence of deficits in the reimbursement of care of patients suffering from a rare disease from studies on individual rare diseases.

**Objectives:**

To investigate current forms of care provision and reimbursement of centres for rare diseases and to develop future approaches for sustainable compensation.

**Materials and methods:**

Initially, centres for rare diseases in Germany were asked to provide information about their forms of care and reimbursement using questionnaires. Subsequently, two focus group interviews and one expert interview with representatives from centres for rare diseases, health insurance, health politics and patients were conducted to discuss current and future meritocratic forms of care provision and reimbursement. The data were evaluated using content analysis.

**Results and conclusions:**

Thirty-nine centres for rare diseases participated in the questionnaire survey. Of those, 38% receive a flat fee/allowance for university outpatient departments, the amount of which varies notably, and 41% obtain a mixed payment comprising an allowance for university outpatient departments and other forms of reimbursement. An under-recovery of costs in centres for rare diseases and its impact on patient care were mentioned in the interviews. In this context, a need to further develop forms of care provision and reimbursement has been identified. Participants prefer a special flat fee/allowance for rare diseases that covers the time-consuming care for patients with rare diseases.

## Einleitung/Hintergrund

In Deutschland leben rund 4 Mio. Menschen mit einer seltenen Erkrankung [1]. Dabei handelt es sich häufig um schwerwiegende und chronische Systemerkrankungen, die einer komplexen Diagnostik und Therapie bedürfen. Um eine hochwertige spezialisierte Versorgung der Betroffenen sicherzustellen, haben sich seit dem Jahr 2009 zahlreiche Zentren für seltene Erkrankungen (ZSE) gebildet [2, 3]. Diesen Zentren kommt im Versorgungsprozess eine zentrale Rolle zu, da sie Kompetenzen und Strukturen bündeln, zielgerichtete Therapieangebote vorhalten und Forschungsaktivitäten vorantreiben.

Um die Zentrenbildung nachhaltig zu fördern, wurde im Rahmen des Nationalen Aktionsplans für Menschen mit seltenen Erkrankungen im Jahr 2013 ein sog. Zentrenmodell etabliert, welches die Strukturen und Aufgaben der ZSE konkretisiert und die interdisziplinäre und multiprofessionelle Versorgung in Typ-A-, -B- und -C-Zentren unterscheidet [4]. An der Spitze der ZSE stehen demnach die Typ-A-Zentren oder „Referenzzentren“, die Menschen mit gesicherter seltener Erkrankung in geeignete Versorgungsstrukturen überführen. Werden Patient:innen mit unklarer Diagnose, aber Hinweisen auf ein seltenes Leiden vorstellig, können diese Verdachtsfälle ebenfalls im Typ-A-Zentrum abgeklärt werden.

Angegliedert an diese übergeordnete Struktur sind mehrere Typ-B-Zentren [5]. Hierbei handelt es sich um Fachzentren, die über ein interdisziplinäres Versorgungsangebot für bestimmte Erkrankungen und Erkrankungsgruppen verfügen. Die Typ-C-Zentren agieren dabei als Kooperationszentren für spezifische Erkrankungen oder Krankheitsgruppen und stellen für Patient:innen mit gesicherter Diagnose oder konkreter Verdachtsdiagnose die ambulante Versorgung sicher. Typ-C-Zentren können bspw. niedergelassene Schwerpunktpraxen, Gemeinschaftspraxen, Medizinische Versorgungszentren (MVZ) oder Krankenhäuser sein.

Eng verknüpft mit der Zentrenbildung ist die Frage der Finanzierung der Versorgung in den Zentren. Die Vergütung der Versorgungsleistungen ist dabei grundsätzlich im Fünften Buch Sozialgesetzbuch (SGB V) geregelt und richtet sich nach der Versorgungsform, auf Grundlage derer die Behandlung der Patient:innen stattfindet. Der Gesetzgeber hat in den vergangenen Jahrzehnten im Rahmen diverser Reformen verschiedene Möglichkeiten zur ambulanten Versorgung im Krankenhaus geschaffen und somit einen Beitrag zur Sicherstellung einer spezialisierten und hochwertigen Behandlung für Patient:innen mit besonders komplexen und schwerwiegenden Erkrankungen geleistet [6]. Jedoch hat sich damit eine große Vielfalt und Heterogenität der Versorgungs- und Vergütungsformen herausgebildet (Tab. 1). Diese sind immer schwieriger zu durchschauen und bedingen teilweise als ungerecht empfundene Unterschiede bei der Vergütung teils identischer oder ähnlicher Leistungen.

**Table 1:** 

§ SGB V	Inhalt
§ 73c a. F. (alte Fassung)	Besondere ambulante ärztliche Versorgung
§ 95	Medizinische Versorgungszentren
§ 116	Ambulante Behandlung durch Krankenhausärzt:innen
§ 116a	Ambulante Behandlung durch Krankenhäuser bei Unterversorgung
§ 116b	Ambulante Spezialfachärztliche Versorgung
§ 117	Hochschulambulanzen
§ 119	Sozialpädiatrische Zentren
§ 119c	Medizinische Behandlungszentren
§ 120 Abs. 1a	Pädiatrische Spezialambulanz
§ 140a	Besondere Versorgung

Ambulanzen, Institute und Abteilungen der Hochschulkliniken, sogenannte Hochschulambulanzen (HSA), können nach § 117 SGB V ermächtigt werden, Patient:innen zu behandeln, die aufgrund der Art, Schwere oder Komplexität ihrer Erkrankung einer Behandlung bedürfen. Zu den relevanten Patientengruppen zählen auch Patient:innen mit seltenen Erkrankungen. Die Hochschulambulanzen erhalten für ihre Versorgungsleistungen je Behandlungsfall eine pauschalierte Vergütung. In Abhängigkeit von der Hochschulambulanz und dem Behandlungsaufwand können für jede Klinik bis zu 50 Pauschalen vereinbart werden, welche die unterschiedlichen Behandlungskosten adäquat abbilden sollen [7].

Auch *Krankenhausärzt:innen*, die über eine abgeschlossene Weiterbildung verfügen, können nach § 116 SGB V zur Teilnahme an der vertragsärztlichen Versorgung ermächtigt werden. Voraussetzung hierfür ist jedoch, dass eine ausreichende Versorgung der Patient:innen ohne die besonderen diagnostischen und therapeutischen Methoden der/des Krankenhausärzt:in nicht anderweitig sichergestellt werden kann. Die Vergütung ist gemäß § 120 SGB V aus dem Budget der Vertragsärzt:innen im niedergelassenen Bereich zu finanzieren und führt somit zu einer Verringerung der vertragsärztlichen Einnahmen. In der Regel werden daher nur sehr begrenzt Ermächtigungen vom Zulassungsausschuss erteilt. Der Umfang der Versorgungstätigkeit ist vertraglich auf spezifische Ziffern des EBM (Einheitlicher Bewertungsmaßstab^1^) festgelegt.

Neben einzelnen Krankenhausärzt:innen können gemäß § 116a SGB V auch *Krankenhäuser im Falle einer Unterversorgung* oder bei lokalen Versorgungsbedarfen eine Ermächtigung zur vertragsärztlichen Versorgung erhalten. Diese gilt für 2 Jahre und entspricht im Hinblick auf den zu gewährenden Versorgungsumfang und die Vergütung der persönlichen Ermächtigung. Gemeinhin wird die Verknüpfung eines Versorgungsauftrags an eine Person gegenüber einer Institution von den Zulassungsausschüssen bevorzugt, um eine hohe Behandlungsqualität sicherzustellen. Dies führt jedoch gleichzeitig zu einer verminderten Kapazität und Flexibilität in der Versorgung, weshalb Kliniken *Institutionsermächtigungen* auch vor dem Hintergrund einer teamorientierten Therapiekoordination bevorzugen.

Des Weiteren ist mit § 116b SGB V die Möglichkeit für die *Ambulante Spezialfachärztliche Versorgung (ASV)* spezifischer seltener Erkrankungen geschaffen worden. Bereits im Jahr 2004 wurden Kliniken für die ambulante Versorgung komplexer Erkrankungen geöffnet. Der Gemeinsame Bundesausschuss (G-BA) wurde damit beauftragt, die seltenen Erkrankungen, Erkrankungen mit besonderen Krankheitsverläufen und hochspezialisierten Leistungen zu konkretisieren, welche ambulant erbracht werden können. Im Rahmen des GKV-Versorgungsstrukturgesetzes 2012 hat der Gesetzgeber den Geltungsbereich um vertragsärztliche Leistungserbringer:innen erweitert. Bislang liegen Konkretisierungen für 8 seltene Erkrankungen vor, darunter Mukoviszidose und Sarkoidose. Die Vergütung der ASV ist einheitlich für Klinik- und Vertragsärzt:innen und richtet sich derzeit nach festen Preisen des EBM bzw. bei Leistungen, die nicht Bestandteil des EBM sind, nach der Gebührenordnung für Ärzte (GOÄ). Langfristig soll eine eigene Vergütungssystematik entwickelt werden.

Ist eine ausreichende Versorgung über Frühförderstellen nicht sichergestellt, können auch *Sozialpädiatrische Zentren (SPZ)* zur ambulanten Behandlung von *Kindern und Jugendlichen* nach § 119 SGB V ermächtigt werden. Die Ermächtigung wird abhängig vom Bedarf zeitlich begrenzt erteilt. Die Grundlage der Leistungsfinanzierung ist in § 120 Abs. 2 SGB V geregelt, der eine Pauschale vorsieht, die unmittelbar von den Krankenkassen vergütet wird.

Ambulanzen, die ausschließlich Kinder versorgen, haben außerdem die Möglichkeit, gemäß § 120 Abs. 1a SGB V als *Pädiatrische Spezialambulanz *vergütet zu werden. Hierfür werden zwischen den Landesverbänden der Krankenkassen und den Ersatzkassen einheitliche einrichtungs- oder fallbezogene Pauschalen vereinbart, die unmittelbar durch die Krankenkassen vergütet werden. Auch *Einrichtungen der Behindertenhilfe* können nach § 119a SGB V vom Zulassungsausschuss zur ambulanten Versorgung von Menschen mit geistiger Behinderung zugelassen werden. Ihre Vergütung ist wie für die Pädiatrischen Spezialambulanzen in § 120 SGB V geregelt und erfolgt anhand einer pauschalierten Vergütung durch die Krankenkassen.

Daneben bieten *Medizinische Versorgungszentren (MVZ)* nach § 95 SGB V auch stationären Leistungserbringer:innen die Möglichkeit, an der ambulanten vertragsärztlichen Versorgung teilzunehmen. Bei MVZ handelt es sich um fachübergreifende ärztlich geleitete Einrichtungen, in denen Ärzt:innen, die in das Arztregister eingetragen sind, als Angestellte oder Vertragsärzt:innen tätig sind. Die Vergütung orientiert sich an der Honorierung anderer ambulanter Leistungserbringer:innen. So bekommt jede/jeder Ärzt:in im MVZ ein individuelles Regelleistungsvolumen (RLV) und ein qualifikationsgebundenes Zusatzvolumen (QZV). Gemeinsam bilden sie das Budget für kassenärztliche Leistungen des MVZ. Des Weiteren können Einzelleistungen nach dem EBM bzw. privatärztliche Leistungen (z. B. IGeL) gemäß der GOÄ abgerechnet werden.

Trotz der vielfältigen gesundheitspolitischen Bemühungen, angemessene Rahmenbedingungen für die ambulante Leistungserbringung für Menschen mit seltenen Erkrankungen durch stationäre Einrichtungen und stationär tätige Ärzt:innen sowie deren Vergütung zu schaffen, liefern Studien Hinweise darauf, dass in ZSE häufig keine kostendeckende Erbringung von Leistungen möglich ist. Dies ist insbesondere darauf zurückzuführen, dass die Vergütungssysteme die Komplexität und den überdurchschnittlichen Versorgungsaufwand seltener Erkrankungen nicht ausreichend abbilden. So konnte beispielsweise eine Untersuchung zur ambulanten Versorgung von Mukoviszidose in Deutschland zeigen, dass nur rund die Hälfte der in den Ambulanzen real anfallenden Kosten durch die Vergütungen der Kostenträger gedeckt werden, wobei die zugrunde liegenden Daten jedoch teilweise lückenhaft waren [8]. Dies kann insbesondere auf den hohen zeitlichen und personellen Aufwand bei der ambulanten Versorgung von Mukoviszidose-Patient:innen in den Zentren zurückgeführt werden. Eine Analyse der Vergütungssituation bei der ambulanten Behandlung nach § 116b SGB V am Beispiel des Marfan-Syndroms konnte ebenfalls eine deutliche Unterdeckung des angefallenen Ressourcenaufwands feststellen [9]. Im Rahmen der Untersuchung wurden ebenfalls die Kosten der medizinischen Behandlung und die Erlössituation verglichen. Es zeigte sich, dass Zentren, die als Hochschulambulanz tätig waren, eine Unterdeckung von 84 % aufwiesen. Bei Zentren, die ihre Patient:innen im Rahmen der ASV versorgten, lag eine deutlich niedrigere Kostendeckungslücke vor. Dennoch wurden auch über die ASV 22 % der Leistungsausgaben nicht von den Krankenkassen vergütet.

Damit eine qualitativ hochwertige Versorgung von Betroffenen dauerhaft sichergestellt und die Versorgungs- und Vergütungsstrukturen weiterentwickelt werden können, sind zunächst Informationen über die aktuelle Istsituation der Versorgungs- und Vergütungslandschaft der B‑Zentren in Deutschland nötig. Im Rahmen der vorliegenden Studie soll daher zunächst quantitativ eruiert werden, auf welcher rechtlichen Grundlage nach SGB V die Leistungserbringung und -vergütung stattfinden. Weiterführende qualitative Untersuchungen zur Angemessenheit der Vergütungsinstrumente aus Sicht von Leistungserbringer:innen und Kostenträgern sollen die bisher kaum untersuchte Thematik der finanziellen Situation der ZSE näher beleuchten. Abschließend werden Ansätze zur Sicherstellung nachhaltiger Versorgungsstrukturen für seltene Erkrankungen und Möglichkeiten einer leistungsorientierten Vergütung der Versorgung von Menschen mit seltenen Erkrankungen an ZSE aufgezeigt.

## Methoden

### Quantitative Erhebung der Versorgungs- und Vergütungsstrukturen in den B‑Zentren (Ist-Situation)

In enger Abstimmung mit verschiedenen Leiter:innen von B‑Zentren aus dem Bereich der genetischen Erkrankungen der Haut, des Auges sowie der angeborenen Störungen des Stoffwechsels in Deutschland wurde ein Fragebogen entwickelt, mit dem die Istsituation der aktuellen Versorgungs- und Vergütungsstrukturen abgebildet werden kann (Abb. Z1 im Onlinematerial). Bei B‑Zentren handelt es sich um Fachzentren, die über ein interdisziplinäres Versorgungsangebot für bestimmte Erkrankungen und Erkrankungsgruppen verfügen. Angegliedert sind diese an Typ-A-Zentren oder Referenzzentren, die u. a. Patient:innen ohne Diagnose, die nicht an einer gesicherten seltenen Krankheit leiden, und Menschen mit gesicherter seltener Erkrankung in geeignete Versorgungsstrukturen überführen. Neben der rechtlichen Grundlage für die Versorgung nach SGB V umfasst der Fragebogen Angaben zur Ausgestaltung der Vergütungsform bei der Erbringung ambulanter Leistungen an B‑Zentren. Der Fragebogen wird komplettiert durch Fragen zu zusätzlichen finanziellen Mitteln (z. B. in Form von Spenden, Stiftungsgeldern, Forschungsmitteln), zu Stärken und Schwächen der bisherigen Versorgungs- und Vergütungsformen, zu aktuell nicht vergüteten Leistungsbereichen sowie zu Ansätzen für zukünftig präferierte Versorgungs- und Vergütungsformen.

Nach einer Vorstellung der geplanten Erhebung bei der Arbeitsgemeinschaft der Zentren für seltene Erkrankungen (AG-ZSE), einem Zusammenschluss der ZSE, der gemeinsame Aktivitäten der Zentren und ihrer Mitarbeitenden koordiniert, erhielten im September 2019 sämtliche B‑Zentren in Deutschland (*N* = 321) per E‑Mail einen Fragebogen. Hierzu wurde eine vom Nationalen Aktionsbündnis für Menschen mit seltenen Erkrankungen (NAMSE) zur Verfügung gestellte Liste zu Ansprechpartner:innen der A‑ und B‑Zentren in Deutschland herangezogen und über eine Handrecherche ergänzt. Da der Rücklauf der Fragebogen insgesamt sehr niedrig ausfiel, wurden sämtliche B‑Zentren, die sich nicht beteiligt hatten, 2 weitere Male mit Bitte um Unterstützung angeschrieben. In den Sitzungen der AG-ZSE stellten die Beteiligten zudem den Projektstand regelmäßig vor und baten um Teilnahme an der Befragung. Nach Abschluss der Erhebung im Dezember 2019 erfolgte eine deskriptive Analyse der Daten.

### Qualitative Erhebung für Ansätze zur Weiterentwicklung der Versorgungs- und Vergütungsstrukturen der ZSE (Soll-Situation)

Neben der Planung und Durchführung der quantitativen Erhebung erfolgte ab Mitte 2019 die Konzeption von Fokusgruppendiskussionen zur Entwicklung nachhaltiger Versorgungs- und Vergütungsstrukturen. Bei Fokusgruppen handelt es sich um ein häufig verwendetes Instrument, um im Rahmen von moderierten und fokussierten Diskussionsrunden die Meinungen und Ideen der Gruppenteilnehmer:innen zu einem vorab definierten Thema einzuholen [10]. Durch den gemeinsamen Austausch und die Notwendigkeit zur Begründung der eigenen Ansichten und Haltungen gegenüber den anderen Teilnehmer:innen kann eine intensivere Auseinandersetzung mit dem diskutierten Thema stattfinden als in Einzelinterviews. Geplant wurde eine Fokusgruppe mit Vertreter:innen der Zentren und eine Gruppe mit gesundheitspolitischen Akteur:innen sowie Mitgliedern der Patientenvertretung. Hierfür wurde zunächst ein Leitfaden entwickelt, der die Beteiligten dazu anregen sollte, über die Heterogenität der aktuellen Versorgungs- und Vergütungsformen sowie die Ansätze zur Weiterentwicklung der Versorgungs- und Vergütungsstrukturen zu diskutieren [11].

Aufbauend auf den vorläufigen Ergebnissen der Fragebogenerhebung fand im November 2019 eine Anpassung des Leitfadens statt, der zudem mit externen Expert:innen in qualitativer Forschung abgestimmt wurde. Nach Durchführung der ersten Fokusgruppendiskussion mit Zentrumsvertreter:innen erfolgte eine Ergänzung des Leitfadens für die zweite Fokusgruppendiskussion. Über bestehende Kontakte, einen Aufruf bei der AG-ZSE-Sitzung, eine Anfrage an die NAMSE-Steuerungsgruppe und die B‑Zentren wurden Teilnehmer:innen für die Fokusgruppen rekrutiert. Im Anschluss an die Fokusgruppendiskussionen wurde eine teilnehmende Person, die kurzfristig nicht an der Fokusgruppendiskussion mitwirken konnte, im Rahmen eines Experteninterviews befragt. Bei Experteninterviews handelt es sich um ein etabliertes Verfahren, bei dem über strukturierte leitfadengestützte Interviews das organisatorische und institutionelle Wissen einer Fachperson erhoben wird. Gegenstand der Forschung sind somit nicht die individuellen Werte und Vorstellungen der interviewten Person, sondern deren spezifisches Fachwissen [12, 13].

Die Fokusgruppendiskussionen und das Experteninterview wurden auf Tonband aufgezeichnet und anschließend transkribiert. Die Auswertung der Transkripte erfolgte inhaltsanalytisch in Anlehnung an Kuckartz unter Entwicklung eines umfangreichen Kategoriensystems mit Unterstützung der Software MAXQDA (Verbi Software, Berlin, Deutschland) [14]. Vor dem Hintergrund der Fragebogenerhebung konnten zunächst verschiedene Kategorien deduktiv erarbeitet werden. Ergänzt wurden diese um Kategorien, die induktiv aus den Fokusgruppendiskussionen und dem Experteninterview abgeleitet werden konnten. Hierzu wurden die Transkripte zunächst im Hinblick auf die Forschungsfragen durchgesehen und Oberkategorien definiert. Diese wurden im Rahmen einer zweiten Durchsicht spezifiziert, sodass ein System aus verschiedenen Ober- und Subkategorien erstellt werden konnte.

## Ergebnisse

### Ist-Situation: Aktuelle Versorgungs- und Vergütungssituation

Insgesamt 39 B‑Zentren aus 8 Bundesländern haben sich an der schriftlichen Erhebung beteiligt, dies entspricht einer Rücklaufquote von 12 % (Tab. 2). In Anlehnung an die Einteilung seltener Erkrankungen nach Organsystemen [15] ist ein Drittel der Zentren auf die Versorgung neuromuskulärer Erkrankungen spezialisiert, weitere 18 % befassen sich mit angeborenen Stoffwechselstörungen. Es folgen Einrichtungen zur Therapie von seltenen Erkrankungen der Haut, Immundefizienzen, Erkrankungen des Auges sowie Mukoviszidose und anderen Lungenerkrankungen. 2 Ambulanzen für Skelettdysplasien sowie jeweils 1 zur Behandlung von seltenen Erkrankungen des Verdauungstraktes und der Blutbildung komplettieren die Stichprobe.

**Table 2:** 

Bundesländer	Anzahl Zentren (*n*)
Bayern	11
Hamburg	3
Mecklenburg-Vorpommern	4
Niedersachsen	4
Nordrhein-Westfalen	6
Sachsen	7
Sachsen-Anhalt	1
Schleswig-Holstein	2
Behandelte Erkrankungsgruppen	Anzahl Zentren (*n*)
Angeborene Störung der Blutbildung	1
Angeborene Störung des Stoffwechsels	7
Genetische Erkrankung des Auges	3
Genetische Erkrankung der Haut	5
Genetische Erkrankung des Verdauungstrakts	1
Immundefizienzen	4
Mukoviszidose und verwandte Lungenerkrankungen	3
Neuromuskuläre Erkrankung	13
Skelettdysplasien	2

15 Zentren (38,46 %) erbringen ihre Leistungen nach § 117 SGB V als Hochschulambulanz und erhalten eine individuell verhandelte HSA-Pauschale. Die Höhe der pauschalierten Vergütung variiert stark zwischen 50 € und 1490 € pro Patient:in und Quartal (p. P./Q.) und liegt bei durchschnittlich 266,10 € (Spannweite 1440 €). Bei 16 Zentren erfolgt die Vergütung einerseits als Hochschulambulanz über eine HSA-Pauschale und andererseits auch über weitere Vergütungsformen. So verfügen 5 ermächtigte Hochschulambulanzen zusätzlich über eine persönliche Ermächtigung von Krankenhausärzt:innen nach § 116 SGB V. Die pauschalierte Vergütung über die Hochschulambulanzpauschale beträgt in diesen Zentren zwischen 110 € und 700 € p. P./Q. (Mittelwert 213 €; Spannweite 510 €). Die Vergütungshöhe der nach EBM abgerechneten Leistungen ist unbekannt, da keine abrechenbaren Ziffern oder beispielhafte Abrechnungen übermittelt wurden. Eine Übersicht zu Versorgungs- und Vergütungsstrukturen der teilnehmenden B‑Zentren findet sich in Tab. Z1 (Onlinezusatzmaterial).

In jeweils einem Zentrum kommt neben der HSA-Ermächtigung und persönlichen Ermächtigungen eine Leistungserbringung nach § 116b SGB V ASV bzw. § 120a SGB V als Pädiatrische Ambulanz zum Tragen. Darüber hinaus berichtet ein Zentrum, zusätzlich zur HSA- und persönlichen Ermächtigung auch eine Ermächtigung des Krankenhauses gemäß § 116a SGB V, eine Anerkennung als Sozialpädiatrisches Zentrum gemäß § 119 SGB V und einer Pädiatrischen Ambulanz nach § 120a SGB V zu besitzen. 2 Zentren rechnen neben der HSA-Pauschale einzelne Leistungen gemäß EBM im Rahmen der ASV ab.

Insgesamt wird eine erhebliche Heterogenität der Versorgungs- und Vergütungsformen deutlich, wobei in der Praxis in den Zentren in vielen Fällen mehrere verschiedene Möglichkeiten der Versorgung an Krankenhäusern angewendet werden und dafür unterschiedliche Vergütungen nach EBM, GOÄ oder Pauschale erfolgen.

Zusätzliche Mittel in Form von Spenden von Pharmaunternehmen und Privatpersonen erhalten 6 B‑Zentren, diese variieren zwischen 1000 € und 60.000 € jährlich. In 7 B‑Zentren stehen darüber hinaus Forschungsgelder zwischen 2000 € und 50.000 € p. a. zur Verfügung. Über Stiftungsgelder zwischen 1000 € und 200.000 € jährlich verfügen 6 Zentren.

### Ist-Situation: Wahrnehmung der finanziellen Situation und bisheriger Vergütungsstrukturen

Im Rahmen der Studie wurden 2 Fokusgruppendiskussionen durchgeführt. In der ersten Diskussionsrunde fanden sich 4 Vertreter:innen verschiedener B‑Zentren und Indikationen teils persönlich, teils per Video- bzw. Telefonzuschaltung zusammen. Die zweite Fokusgruppe bestand aus 9 Teilnehmer:innen, zu denen Vertreter:innen aus der Gesundheitspolitik, von Patienten- und Klinikverbänden und der Pharmaindustrie gehörten. Auch bei dieser Gruppe gab es neben persönlichen Teilnahmen zusätzliche virtuelle bzw. telefonische Zuschaltungen. Ein Experteninterview mit der Vertretung einer Krankenkasse komplettierte die Stichprobe. Ausgewählte Zitate aus den Fokusgruppen und dem Experteninterview zur Untermauerung der qualitativen Ergebnisse finden sich in Tab. Z2 (Onlinematerial).

Die Frage nach der Angemessenheit der Vergütung wird von den Teilnehmer:innen unterschiedlich diskutiert. Die Vertreter:innen der Gesundheitspolitik und der Kostenträger weichen einer expliziten Stellungnahme tendenziell aus oder verweisen auf den geringen Anteil der seltenen Erkrankungen an den Gesamtkosten eines Klinikums. Demgegenüber liegt aus Sicht der befragten Zentrenvertreter:innen bei vielen Indikationen eine mangelnde Kostendeckung bei der ambulanten Versorgung vor, welche die B‑Zentren vor erhebliche Herausforderungen stellt. Interne Berechnungen der Kliniken spiegeln wider, dass die Ressourcenverbräuche für die erbrachten Leistungen nicht durch die Erlöse aus den verschiedenen Versorgungs- und Vergütungsformen gedeckt sind. Eine konkrete Einsicht in entsprechende Berechnungen wurde im Rahmen der Studie jedoch nicht gewährt.

Die Berechnungen der Kliniken, welche die Defizite in der Vergütung aufzeigen, sind gemäß der Zentrenvertreter:innen jedoch nicht geeignet, um eine stärker an den Aufwendungen oder Leistungen orientierte Vergütung mit den Krankenkassen auszuhandeln. Dies liegt nach Aussage der Kliniker:innen nicht an der Qualität der Daten, sondern an der mangelnden Bereitschaft aufseiten der Kostenträger, diese als Verhandlungsgrundlage anzuerkennen. Auch ein konzertiertes Vorgehen durch Zusammenschlüsse der B‑Zentren auf Landesebene konnte bislang keine nachhaltigen Veränderungen der Vergütungssituation erwirken. Seitens vieler Zentrenvertreter:innen werden die Verhandlungen auf Ebene der Bundesländer als vergeblich wahrgenommen. Um die Situation langfristig zu verbessern, seien Petitionen über den Bundestag notwendig, um eine Verankerung im Gesetz zu erreichen. Auch Rahmenregelungen auf Bundesebene wären sinnvoll.

Nach Einschätzungen der teilnehmenden Zentren- und Patientenvertreter:innen hat die Vergütungssituation bereits Auswirkungen auf die Patientenversorgung und mindert nachhaltig die Bereitschaft, sich in der Versorgung von Menschen mit seltenen Erkrankungen zu engagieren und entsprechende Strukturen aufzubauen und zu erhalten. Kostenträger und gesundheitspolitische Vertreter:innen hingegen sehen grundsätzlich keine spürbaren Einschränkungen für die Versorgung von Patient:innen.

Die aus Sicht der B‑Zentren vorliegende unzureichende Finanzierung führt zu Unzufriedenheit und Frustration. Dabei zeigt sich die Unzufriedenheit mit den Vergütungsstrukturen über verschiedene Versorgungsansätze hinweg.

Viele der Zentren werden ausschließlich oder in Kombination mit anderen Versorgungsformen über die HSA-Pauschale vergütet. Insbesondere die für die Versorgung der seltenen Erkrankungen bedeutsame interdisziplinäre Zusammenarbeit und der erhöhte Zeitaufwand bei der Versorgung von Menschen mit seltenen Erkrankungen werden aktuell nicht ausreichend abgebildet. Zum einen handelt es sich bei den vereinbarten Pauschalen häufig um eine nicht auf die Besonderheiten der seltenen Erkrankungen ausgelegte Pauschale, sondern um eine für alle HSA-Patient:innen eines Klinikums einheitlich verhandelte Vergütung. Zum anderen verbleibt die HSA-Pauschale meist dort, wo Patient:innen als Erstes vorstellig werden. Leistungen anderer Klinikbereiche, wie z. B. Laboruntersuchungen, können hierüber häufig nicht abgerechnet werden. Dies liegt zum Teil auch daran, dass der pauschalierte Betrag zu gering ist, um ihn auf verschiedene Einrichtungen aufzuteilen. Zwar konnten in den vergangenen Jahren Verbesserungen in der Höhe der HSA-Pauschale erreicht werden, diese entsprechen jedoch nicht dem tatsächlichen Ressourcenaufwand der Kliniken.

Auch die u. a. speziell für die Versorgung von seltenen Erkrankungen geschaffene ASV-Regelung wird aus Sicht der Teilnehmenden kritisch betrachtet und weist insbesondere Schwächen im komplexen Anzeigeverfahren auf. Auch kann der Erfahrung einiger Kliniker:innen nach die Versorgung mancher Indikationen im Rahmen der ASV nicht hinreichend vergütet werden.

Einige der Zentrumsvertretungen verfügen zudem über Erfahrungen mit persönlichen Ermächtigungen. Diese werden kritisch diskutiert. Während einzelne, im Rahmen der Ermächtigung festgelegte Leistungen erbracht und vergütet werden können, bleiben darüber hinausgehende unberücksichtigt. Ebenso werden die Abhängigkeit von der Ermächtigungserteilung durch den Zulassungsausschuss bzw. die Konflikte mit den Niedergelassenen und die damit verbundene mangelnde Planungssicherheit kritisiert.

Aufgrund der herausfordernden Vergütungssituation über die gesetzlich verankerten Versorgungsmöglichkeiten sind die Zentren mitunter auf weitere Finanzmittel angewiesen. Zum Teil können Forschungsgelder zur Querfinanzierung bestimmter Leistungen eingesetzt werden. Gleichzeitig machen die Zentren, die über viele Forschungsmittel verfügen, die Erfahrung, dass ihnen weniger Finanzmittel vom Klinikum zugeteilt werden, was zu einer eingeschränkten Planungssicherheit nach Auslaufen der Forschungsprojekte führt.

Insgesamt ist festzustellen, dass von den Zentren über verschiedene vorhandene Versorgungs- und Vergütungsformen hinweg eine Unterdeckung der Kosten für die Versorgung von seltenen Erkrankungen berichtet wird. Die Vergütungsdefizite zeigten bereits Auswirkungen auf die Bereitschaft zum Erhalt spezialisierter Versorgungsstrukturen. Ein Bedarf zur Weiterentwicklung der Versorgungs- und Vergütungsstrukturen, um die Versorgung von Betroffenen nachhaltig sicherstellen zu können, wird ausdrücklich benannt.

### Soll-Situation: Ansätze für zukünftige Versorgungs- und Vergütungsstrukturen

Um eine qualitativ hochwertige Versorgung und angemessene Vergütung von ZSE nachhaltig sicherstellen zu können, wäre aus Perspektive der B‑Zentren eine einheitliche und leistungsorientierte gesetzliche Regelung der Vergütung ambulanter Leistungen in ZSE notwendig. Die vom G‑BA beschlossenen bundeseinheitlichen Qualitätsanforderungen für die Übernahme von besonderen Aufgaben durch Einrichtungen der Spitzenmedizin und die Zentrumszuschläge stellten vorwiegend auf die stationäre Versorgung sowie zentrumsferne Leistungen für andere Einrichtungen ab. Eine Verbesserung der Vergütungssituation für die B‑Zentren könne aus Sicht der Teilnehmenden dadurch nicht erreicht werden. Die fehlende Grundlage im SGB V führe ferner dazu, dass die Kostenträger ihrer im NAMSE-Prozess eingegangenen Verpflichtung zur Finanzierung der Zentren nicht nachkommen.

Aus Sicht der Teilnehmenden sollte eine vereinheitlichte Versorgungs- und Vergütungsstruktur angestrebt werden, die auf die besonderen personellen und zeitlichen Aufwendungen, die die Behandlung der seltenen Erkrankungen mit sich bringt, fokussiert.

Dabei werden die zeitliche Komponente und die damit verbundenen hohen Personalkosten übergreifend als wesentlicher Faktor für die überdurchschnittlichen Kosten der Versorgung seltener Erkrankungen verstanden. Kennzeichnend für die ZSE sei eine „Vorhaltemedizin“, die wenig Patient:innen bei gleichzeitiger Vorhaltung aufwendiger Diagnostik und Therapie durch hochspezialisierte Leistungserbringer:innen bedeute. Ebenfalls handele es sich um eine „sprechende Medizin“, bei der die Versorger:innen viel Zeit für die Kommunikation mit Patient:innen, Kolleg:innen und anderen an der Versorgung beteiligten Leistungserbringer:innen benötigen. Bei der Vergütung seltener Erkrankungen müssten diese zeitlichen Aufwendungen angemessen berücksichtigt werden.

Aufgrund der Erfahrungen der Kliniken aus den Vergütungsverhandlungen mit den Krankenkassen wird der Einsatz detaillierter Kostennachweise als Verhandlungsgrundlage kritisch diskutiert. Eine Darstellung der zeitlich aufgewendeten Ressourcen erscheint dagegen als sinnvolle Alternative, um den zusätzlichen zeitlichen Aufwand der Zentren abbilden zu können. Nach Ansicht der Kliniken verfügen die Zentren über die entsprechenden Informationen, um den Krankenkassen den personellen Aufwand sowie die Kontaktzeiten darlegen zu können. Auch aus Sicht des Kostenträgers wird eine solche leistungsbezogene Verhandlungsgrundlage präferiert.

Als besonders herausfordernd bezüglich der Herausarbeitung nachhaltiger Vergütungsansätze wird auch die Abwägung zwischen dem bürokratischen Aufwand und der Genauigkeit der Vergütung erachtet. Grundsätzlich teilen die Teilnehmenden die Auffassung, dass auch eine pauschalierte Vergütung das Risiko überhöhter oder zu niedriger Vergütungen beinhaltet, aber einen guten Kompromiss im Sinne einer „Mischfinanzierung“ für die unterschiedlich aufwendig zu versorgenden Fälle darstellt. Die Vergütung der Leistungen in pauschalierter Form wird mehrheitlich präferiert, da die Erarbeitung einer eigenen Vergütungssystematik für die ambulante Versorgung an Krankenhäusern in Anlehnung an das DRG-System^2^ – wie es in der ASV-Regelung vom Grundsatz her vorgesehen ist – aus administrativen Gründen als nicht realistisch eingeschätzt wird.

Als eine Möglichkeit wird die Etablierung einer „Sonderpauschale für seltene Erkrankungen“ angeführt. Die Loslösung einer pauschalierten Vergütung für seltene Erkrankungen von der HSA-Pauschale hätte auch für B‑Zentren, die nicht an ein Universitätsklinikum angegliedert sind, den Vorteil, diese zusätzlich abrechnen zu können. Aber auch eine differenziertere HSA-Pauschale, die gesondert nach Gruppen von Indikationen eine leistungsbezogene Vergütung ermöglicht, wird von den Teilnehmenden als positiv beurteilt.

Um verschiedene Schweregrade differenziert abbilden zu können, wird eine abgestufte Pauschale diskutiert. Hierdurch könnte der Anreiz für Krankenhäuser gemindert werden, vorwiegend leichte Fälle zu behandeln. Alternativ wird die Möglichkeit angesprochen, eine über verschiedene Schweregrade gewichtete Pauschale zu ermitteln.

Insgesamt ist ein erheblicher Bedarf an einer von den Zentrumsregelungen losgelösten Weiterentwicklung der ambulanten Vergütungsstrukturen festzustellen. Besonders berücksichtigt werden sollten hierbei die überdurchschnittlich hohen zeitlichen und personellen Aufwendungen in der Versorgung von Betroffenen. Eine Sonderpauschale für seltene Erkrankungen, die diese Besonderheiten einbezieht, wird als Möglichkeit für eine nachhaltige und leistungsorientierte Vergütungsform präferiert.

## Diskussion

Mit der in der Studie durchgeführten quantitativen Erhebung zur Finanzierung der B‑Zentren wurde erstmals angestrebt, eine flächendeckende Erfassung der Versorgungs- und Vergütungsstrukturen der ZSE in Deutschland vorzunehmen. Trotz der geringen Teilnahmequote der Zentren konnte eine sehr heterogene Versorgungs- und Vergütungslandschaft nachgewiesen werden. So konnte gezeigt werden, dass der Großteil der B‑Zentren ihre Patient:innen als Hochschulambulanz versorgt, die eine Pauschale als Vergütung erhält. Ferner konnten zahlreiche Mischformen von HSA-Pauschalen mit weiteren Vergütungsarten bei anderen Versorgungsformen festgestellt werden. Insgesamt lässt sich darüber hinaus eine große Varianz in der Höhe der mit den Kostenträgern ausgehandelten Pauschalen erkennen. Da sich einige ZSE nicht beteiligt haben, kann jedoch nicht abgeschätzt werden, wie sich die Varianz innerhalb spezifischer Indikationen darstellt. Auch Aussagen über zusätzlich vergütete Leistungen sind aufgrund fehlender Angaben zu abrechenbaren EBM-Ziffern nicht möglich.

Die Ergebnisse der inhaltsanalytischen Auswertungen der Fokusgruppendiskussionen und des Experteninterviews belegen, dass die derzeitige Vergütung insbesondere von den Zentren und den Klinikverbünden als unzureichend wahrgenommen wird. Besonders die für die Versorgung von seltenen Erkrankungen notwendigen Vorhaltungen an spezialisiertem Personal und aufwendiger Diagnostik und Therapie würden über die aktuellen Vergütungsstrukturen nicht hinreichend abgebildet. Diese Wahrnehmung stimmt mit vorherigen Untersuchungen zur Erlössituation am Beispiel spezifischer Indikationen überein [8, 9]. Wenngleich das Ausmaß der mangelnden Kostendeckung von Politik, Versorger:innen und Kostenträgern unterschiedlich diskutiert wird, wird ein Handlungsbedarf übergreifend erkannt und die Notwendigkeit für alternative Finanzierungswege gesehen. Die Mehrheit der Teilnehmenden teilt dabei die Auffassung, dass die verschiedenen im Gesetz verankerten Versorgungs- und Vergütungsstrukturen nicht zur nachhaltigen Vergütung der B‑Zentren geeignet sind. So wird die ASV u. a. als zu kompliziert im Anzeigeverfahren und nicht kostendeckend bewertet. Dies deckt sich mit Untersuchungen zur Umsetzung der ASV, die belegen, dass die ASV-Regelungsdichte ein bürokratisches Hindernis darstellt und das Potenzial als innovatives Versorgungskonzept nicht entfaltet hat [16, 17]. Obwohl im Bereich der HSA-Pauschale aus Sicht der Kliniken in den vergangenen Jahren deutliche Verbesserungen in der Vergütung erzielt werden konnten, ist auch diese nach Aussage der ZSE noch nicht kostendeckend.

Grundsätzlich präferieren die Befragten im Hinblick auf zukünftige Vergütungsformen eine pauschalierte Vergütung. In Anbetracht der Vielzahl verschiedener seltener Erkrankungen wird die Entwicklung einer eigenen Vergütungssystematik als unrealistisch und unter administrativen Aspekten als zu aufwendig eingeschätzt. Eine Sonderpauschale für seltene Erkrankungen, welche Versorger:innen zusätzlich zu ihrer regulären Vergütung erhalten, bzw. eine Erhöhung der HSA-Pauschale wird daher als positiv angesehen. Um die überdurchschnittlichen personellen, diagnostischen und therapeutischen Aufwendungen entsprechend abbilden zu können, soll dem Faktor Zeit bei der Kalkulation der Pauschale eine besondere Bedeutung zukommen. Eine Fokussierung auf zeitliche Aufwendungen und erbrachte Leistungen wird auch von Kostenträgerseite präferiert. Die indikationsspezifische Abstaffelung nach verschiedenen Schweregraden wird als sinnvoll erachtet, um den Zentren keine Anreize zu setzen, die Versorgung leichter Fälle vermehrt in den Fokus zu rücken.

Diese Studie unterliegt verschiedenen Limitationen. Zum einen konnte im Rahmen der Fragebogenerhebung nur ein geringer Rücklauf erzielt werden. Grundsätzlich wäre es möglich, dass der Rücklauf im Zusammenhang mit einer Unzufriedenheit der B‑Zentren bzgl. der Vergütungssituation steht und eine geringe Beteiligung Ausdruck für eine überwiegende Zufriedenheit mit den derzeitigen Strukturen ist. Die Autor:innen führen die niedrige Rücklaufquote jedoch vielmehr auf knappe zeitliche Ressourcen, auf die Priorität anderer Thematiken im Versorgungsalltag und insbesondere auf eine mangelnde Bereitschaft, sensible Kostendaten zu Forschungszwecken zur Verfügung zu stellen, zurück. Wie die Analyse der Fokusgruppen und des Interviews gezeigt hat, werden die Vergütungen der Krankenkassen in der Regel innerhalb des Klinikums umverteilt und den Zentren nach Bedarf zugewiesen. Es scheint daher plausibel, dass entsprechende Informationen beim Klinikum verbleiben sollen. Darüber hinaus ist die Höhe der mit den Kassen verhandelten Vergütung das Ergebnis klinikinterner Verhandlungen, was ebenfalls zu einer eingeschränkten Bereitschaft der Fragebogenteilnahme geführt haben könnte. Die Ergebnisse geben somit einen Ausschnitt der Versorgungs- und Vergütungslandschaft wieder, ohne diese repräsentativ darstellen zu können.

Für die Fokusgruppendiskussionen und das Experteninterview liegen ebenfalls Limitationen hinsichtlich der Repräsentativität vor, da im Rahmen qualitativer Untersuchungen grundsätzlich keine repräsentativen Stichproben gezogen werden können. Daher wird durch theoretisches Sampling versucht, eine Reichhaltigkeit und Konsistenz der Ergebnisse zu erzeugen. Hierfür finden gleichzeitig mit der Datenerhebung erste Datenauswertungen statt, die Hinweise darauf geben, welche weiteren relevanten Fälle ausgewählt werden sollten, um den zu untersuchenden Gegenstandsbereich möglichst umfassend abbilden zu können. Das Ziel liegt somit nicht im Produzieren von Ergebnissen, die für eine breite Population repräsentativ sind, sondern darin, zuverlässige und gültige Konzepte zu entwickeln.

## Fazit und Ausblick

Insgesamt kann festgehalten werden, dass eine erhebliche Heterogenität der Versorgungs- und Vergütungsstrukturen in den ZSE vorliegt. Um die spezialisierte Versorgung langfristig sicherzustellen, sollten eine „Sonderpauschale für sE“ sowie die notwendigen Rahmenbedingungen im Gesundheitswesen entwickelt werden. Dies ist auch aus versorgungspolitischer Sicht notwendig, um die Bereitschaft von Ärzt:innen und Einrichtungen, sich nachhaltig im Bereich sE zu engagieren, aufrechtzuerhalten. Es bedarf weiterer Untersuchungen zu einzelnen Leistungen und notwendigen Zeitaufwendungen am Beispiel ausgewählter Indikationen, um sich einer von den Teilnehmenden präferierten Pauschale anzunähern. Die teilnehmenden Leistungserbringer:innen und Kostenträger haben bereits ihre Bereitschaft zur weiteren Zusammenarbeit und Unterstützung erklärt und mögliche Erkrankungen definiert. Zukünftig sollen deshalb modellhaft indikationsspezifische Rahmenvereinbarungen einer leistungsgerechten Vergütung erarbeitet und verhandelt werden. Hierdurch könnte ein innovatives Vergütungskonzept angestoßen werden, welches die Versorgung von Menschen mit seltenen Erkrankungen nachhaltig sicherstellt.

## Supplementary Information




## Footnotes

1 Vergütungssystem der vertragsärztlichen bzw. vertragspsychotherapeutischen Versorgung in Deutschland.

2 Klassifikationssystem für ein pauschaliertes Abrechnungsverfahren nach diagnosebezogenen Fallgruppen (Diagnosis Related Groups – DRG).
