# Supplementary material for: Sustainable reimbursement of the B‑centres for rare diseases in Germany—status quo and solution approaches
Source: Bundesgesundheitsblatt Gesundheitsforschung Gesundheitsschutz. 2022 Jul 21;65(9):872–80. [Article in German] doi: 10.1007/s00103-022-03562-7 (PMC9436832; doi:10.1007/s00103-022-03562-7)
Supplement: Supplementary file 1 [file 103_2022_3562_MOESM1_ESM.pdf]

Bundesgesundheitsblatt – Gesundheitsforschung – Gesundheitsschutz, Ausgabe 9/2022

Onlinematerial zum Beitrag:

Nachhaltige Vergütung der B-Zentren für Seltene Erkrankungen in Deutschland – Status quo und Lösungsansätze

Svenja Litzkendorf[1], Daniela Eidt-Koch[2], Jan Zeidler[1], J.-Matthias Graf von der Schulenburg[1]

[1]Center for Health Economics Research Hannover (CHERH), Leibniz Universität Hannover, Hannover, Deutschland

[2]Fakultät Gesundheitswesen, Ostfalia Hochschule für angewandte Wissenschaften, Wolfsburg, Deutschland

Korrespondenzadresse:

Svenja Litzkendorf

Leibniz Universität Hannover

Center for Health Economics Research Hannover (CHERH)

Otto-Brenner-Str. 7

30159 Hannover

sl@ivbl.uni-hannover.de

Inhalt:

Abbildung Z1: Fragebogen zu Kosten und Finanzierung von B-Zentren für seltene Erkrankungen in Deutschland

Tabelle Z1: Versorgungs- und Vergütungsstrukturen der teilnehmenden B Zentren

Tabelle Z2: Zitate aus den Fokusgruppen und dem Experteninterview

Abbildung Z1: Fragebogen zu Kosten und Finanzierung von B-Zentren für seltene Erkrankungen in Deutschland

Projekt „Kosten und Finanzierung von Zentren für seltene Erkrankungen in Deutschland“

Sehr geehrte Damen und Herren,

im Rahmen unseres Projekts „Kosten und Finanzierung von Zentren für seltene Erkrankungen in Deutschland“ möchten wir einen **Überblick über den aktuellen Stand der Abrechnungsarten und Vergütungsformen in den Zentren für seltene Erkrankungen** in Deutschland erhalten.

Im Nachfolgenden finden Sie eine Auflistung von Versorgungsformen und Abrechnungsarten, nach denen Leistungen, die ambulant in Zentren für seltene Erkrankungen erbracht werden, erbracht und vergütet werden können. Bitte geben Sie jeweils für Ihr B-Zentrum an, **auf Grundlage welcher Paragrafen und in welcher Form Leistungen vergütet werden**. Bitte antworten Sie möglichst ausführlich und nutzen ggf. auch die Freitextfelder. Um einen Überblick über die tatsächlich abrechenbaren Leistungen zu erhalten, wäre es schön, wenn Sie eine beispielhafte Abrechnung bzw. eine Liste der abrechenbaren EBM-Ziffern (und ggf. weitere Ziffern) beifügen könnten. Sollten spezifische Abrechnungsarten und Vergütungsformen fehlen, nach denen in Ihrem B-Zentrum Leistungen erbracht und abgerechnet werden können, bitten wir Sie um Ergänzung.

Vielen Dank für Ihre Unterstützung.

Angaben zur Einrichtung

Name des Ausfüllenden	
Emailadresse	
Telefonnummer	
Name A-Zentrum	
Name B-Zentrum	

Frage 1.) Bitte kreuzen Sie an, auf welcher Grundlage Leistungen in Ihrem B-Zentrum erbracht werden. (Mehrfachnennungen möglich)		
Im ambulanten Bereich		
a)	☐	§ 117 SGB V<br>Hochschulambulanz
b)	☐	§ 116 SGB V<br>Persönliche Ermächtigung von Krankenhausärzten
c)	☐	§ 116a SGB V<br>Institutsbezogene Ermächtigung
d)	☐	§ 116b SGB V<br>Ambulante Spezialfachärztliche Versorgung (ASV)
e)	☐	§ 120 SGB V Abs. 1a<br>Pädiatrische Spezialambulanz
f)	☐	sonstige (bitte erläutern)
Im stationären Bereich		
g)	☐	§ 39 SGB V<br>Teilstationäre Behandlung
h)	☐	sonstige (bitte erläutern)

Frage 2.) Bitte kreuzen Sie an, wie die entsprechenden Leistungen vergütet werden.	Bei Mehrfachnennungen zu Frage 1.) bitte hier ergänzen (a-h)
Im ambulanten Bereich	
☐ ausschließlich auf Basis von EBM-Ziffern	
☐ ausschließlich über eine Pauschale in Höhe von ______ €, diese gilt<br>☐ pro Quartal und Patient<br>☐ (bitte erläutern)	
☐ teilweise auf Basis von EBM-Ziffern und teilweise über eine Pauschale in Höhe von ______ €, letztere gilt<br>☐ pro Quartal und Patient<br>☐ (bitte erläutern)	
☐ auf Basis von GOÄ-Ziffern	
Im stationären Bereich	
☐ ausschließlich auf Basis von DRGs	
☐ teilweise auf Basis von DRGs und teilweise über eine Pauschale in Höhe von ______ €, letztere gilt<br>☐ pro Quartal und Patient<br>☐ (bitte erläutern)	
☐ auf folgende Weise (bitte erläutern):	

Frage 3: Bitte geben Sie uns aus Ihrer Sicht Hinweise für eine mögliche Verbesserung der Vergütung der Versorgung der Sie betreffenden seltenen Erkrankungen an einem Zentrum.
Worin sehen Sie Stärken und Schwächen in den bestehenden Abrechnungsmöglichkeiten?<br>Bitte erläutern:
Welche Leistungsbereiche sind aus Ihrer Sicht nicht ausreichend finanziert?<br>Bitte erläutern:
Wie könnte Ihrer Meinung nach die Ausgestaltung der Vergütungsmöglichkeiten verbessert werden?<br>Bitte erläutern:

Frage 4: Erhält Ihr B-Zentrum über die Vergütungen (siehe Frage 1+2) hinaus weitere finanzielle Mittel speziell für die Versorgung von Patienten?	
☐ Zuschüsse	€ pro Jahr durch:
☐ Spenden	€ pro Jahr durch:
☐ Forschungsmittel	€ pro Jahr durch:
☐ Stiftungsgelder	€ pro Jahr durch:
☐ Sonstige	€ pro Jahr durch:
Bitte erläutern Sie uns im Folgenden, woher diese Mittel stammen, wie diese eingesetzt werden, ob diese zweckgebunden und/oder zeitlich befristet sind.	

Allgemeine Anmerkungen oder Ergänzungen zum Fragebogen bzw. zur Vergütungssituation an Zentren für seltene Erkrankungen:

Vielen Dank für Ihre Unterstützung!

Tabelle Z1: Versorgungs- und Vergütungsstrukturen der teilnehmenden B Zentren

Versorgungs- und Vergütungsgrundlage gemäß SGB V	Vergütungsgrundlage	Anzahl Zentren
§ 117 SGB V Hochschulambulanzen	Pauschale	15
§ 117 SGB V Hochschulambulanzen; § 116 SGB V Ambulante Behandlung durch Krankenhausärzte	GOÄ, EBM, Pauschale	5
§ 117 SGB V Hochschulambulanzen; § 116 SGB V Ambulante Behandlung durch Krankenhausärzte; § 116b SGB V Ambulante Spezialfachärztliche Versorgung	EBM, Pauschale	1
§ 117 SGB V Hochschulambulanzen; § 116 SGB V Ambulante Behandlung durch Krankenhausärzte; § 120a SGB V Pädiatrische Ambulanz	EBM, Pauschale	1
§ 117 SGB V Hochschulambulanzen; § 116 SGB V Ambulante Behandlung durch Krankenhausärzte; § 120a SGB V Pädiatrische Ambulanz; § 116a Ambulante Behandlung durch Krankenhäuser; § 119 SGB V Sozialpädiatrische Zentren	EBM, Pauschale	1
§ 117 SGB V Hochschulambulanzen; § 115b SGB V Ambulantes Operieren	Pauschale	1
§ 117 SGB V Hochschulambulanzen; § 116a SGB V Ambulante Behandlung durch Krankenhäuser; 116b SGB V Ambulante Spezialfachärztliche Versorgung	EBM, Pauschale	1
§ 117 SGB V Hochschulambulanzen; § 140a Besondere Versorgung	GOÄ, EBM	1
§ 117 SGB V Hochschulambulanzen; 119c SGB V Medizinische Behandlungszentren	GOÄ, EBM, Pauschale	1
§ 117 SGB V Hochschulambulanzen; § 116b Ambulante Spezialfachärztliche Versorgung	EBM, Pauschale	2
§ 117 SGB V Hochschulambulanzen; § 116b Ambulante Spezialfachärztliche Versorgung; § 120a SGB V Pädiatrische Ambulanz	EBM, Pauschale	1
§ 117 SGB V Hochschulambulanzen; Fallpauschalen mit Krankenkassen	Pauschale	1
§ 116 SGB V Ambulante Behandlung durch Krankenhausärzte	EBM, Pauschale	2
§ 116b SGB V Ambulante Spezialfachärztliche Versorgung	EBM, Pauschale	1
§ 119 SGB V Sozialpädiatrische Zentren	Pauschale	1
§ 73c SGB V a.F. Besondere ambulante ärztliche Versorgung	Pauschale	2
§ 95 SGB V Medizinische Versorgungszentren	k. A.	2

Tabelle Z2: Zitate aus den Fokusgruppen und dem Experteninterview

Themenbereich	Interviewkontext	Zitat
Angemessenheit der Vergütung	FG 1, B-Zentrum	*„Und dem Ganzen dann 120€ gegenüberstellen, was sie wirklich […] bekommen, dann wissen Sie, dass Sie ungefähr 1000€ drauflegen pro Quartal, pro Patient und dann wissen Sie, dass das irgendwie ein Problem ist.“*
	FG 2, Klinikverband	*„(…) dann haben wir ein Finanzierungssystem, was natürlich diese besonderen Aufwendungen, die wir dort haben, in keinster Weise würdigt.“*
Bisherige Bemühungen, um höhere Vergütung zu erreichen	FG 1, B-Zentrum	*„Aber ich kann Ihnen sagen, dass mit Hilfe des [Name Bundesland] Wissenschaftsministeriums und des Landtages gab es ein Gespräch zur [Name Erkrankung]-Finanzierung in [Name Bundesland], mit allen Uniklinika, mit einer klaren Kostenkalkulation, wo die Krankenkassen klar gesagt haben „Niente. Punkt“. Also alle Kalkulation und alle Überlegungen, was man so machen kann, sind für den Mülleimer.“*
	FG 1, B-Zentrum	*„Egal was Sie machen, reicht nicht aus. Das ist genau das große Problem, was Sie haben. Und die einzige Chance, die ich sehe, sind jetzt richtige Gesetzeslücken oder Schwachsinn im Gesetz zu entdecken und über den Bundestag zu gehen. Alles andere funktioniert nicht.“*
Auswirkungen auf die Versorgung	FG 2, Patientenvertretung	*„Also da haben wir jetzt in den letzten Jahren wirklich Erfahrungen gemacht, dass das zu Problemen in der Versorgung führt.“*
	FG 1, B-Zentrum	*„Also wer will denn jetzt noch B-Zentrum sein, wenn er noch mehr Arbeit macht, für die er kein Geld kriegt?“*
	I 3, Kostenträger	*„Also ich glaube wirklich, wenn die Patienten nicht versorgt wären, dann würde das schon ganz anders kommuniziert werden. Natürlich, wenn man Verbände fragt, Krankenhausverbände oder Krankenhausbetroffene, natürlich sagen die das (…). Ich glaube, man muss das richtig einordnen.“*
Frustration über unzureichende Versorgung	FG 1, B-Zentrum	*„Das machen wir schon seit Jahren, dass wir kalkulieren und uns jedes Mal ärgern und sehen, was das für Defizite ergibt, wenn man das dann der eigentlichen Bezahlung gegenüberstellt.“*
Herausforderungen durch HSA Pauschale	FG 2, Klinikverband	*„Wir haben viele Standorte, die leben noch mit so einer Einheitspauschale. Die kriegen jetzt zurzeit 30 Euro pro Fall. Das betrifft dann den ZSE-Fall genauso wie den Onko-Fall, das betrifft genauso den dermatologischen Fall wie den Fall der Augenambulanz.“*
	FG 1, B-Zentrum	*„Die Zentren arbeiten natürlich nach ihren eigenen Modellen. Viele eben auch über die Hochschulambulanzpauschale, die in keinster Weise den Komplexitätsgrad der Erkrankungen*

		abdeckt. Und den damit verbundenen Aufwand. Und eben auch die interdisziplinäre Kooperation, die eben oft erforderlich ist.“
	FG 2, Klinikverband	*„Und das wäre natürlich im universitären Bereich die Frage der Hochschulambulanzfinanzierung, allerdings ist es ja auch hier so, dass man sagen kann, dass das, was in den Hochschulambulanzen jetzt finanziert wird, bei weitem noch nicht kostendeckend ist.“*
Wahrnehmung der ASV	I 3, Kostenträger	*„Also ASV, finde ich, ist äußerst problematisch. Also (…), ich habe jetzt die dritte oder vierte ASV-Regelung erlebt und die wird immer komplizierter.“*
	FG 1, B-Zentrum	*„Wir rechnen das nach §116b ab und es ist natürlich auch nicht an allen Fällen, grade bei der Betreuung von [Name Erkrankung]-Patienten, ist es auch nicht immer kostendeckend.“*
Erfahrungen mit persönlicher Ermächtigung	I 3, Kostenträger	*„Also Ermächtigung ist schwierig, weil man abhängig ist von der Politik der jeweiligen KV. Das ist nicht ganz einfach, da muss man auch sagen, das ist ein Thema, das wollen die Niedergelassenen wahrscheinlich nicht so gerne haben.“*
Weitere Finanzierungsformen	FG 1, B-Zentrum	*„Wir haben eine Reihe von klinischen Studien, über die wir auch unsere Mitarbeiter finanzieren, wo sich dann manchmal Synergieeffekte auch für die klinische Versorgung der allgemeinen Patienten ergeben.“*
	FG 1, B-Zentrum	*„Sprich, wenn man genug Projekte hat, die einem alles finanzieren oder vieles finanzieren, dann kriegt man halt kein Geld aus diesem Topf. Wie das dann ist, wenn diese Projekte wegfallen, das wird dann möglicherweise schwierig. Das wissen wir nicht. Wir wissen auch nicht was nächstes Jahr kommt.“*
Einschätzung der Zentrumszuschläge	FG 2, Gesundheitspolitik	*„Also es geht ausschließlich um die Finanzierung zusätzlicher, besonderer Aufgaben, die in Zentren wahrgenommen werden, die im Grunde nicht patientenbezogen sind. Also diese Frage der Unterfinanzierung von Leistungen seltener Erkrankungen, sofern Sie der Auffassung sind, dass sie unterfinanziert sind, die wird mit dieser Zuschlagsregelung nicht gelöst.“*
Notwendigkeit einer einheitlichen und leistungsorientierten gesetzlichen Regelung	I 3, Kostenträger	*„Also wir leben ja im ambulanten Krankenhausbereich in einer völlig unsystematischen gesetzlichen Regulierung. Also (…) das muss alles mal systematisch geordnet werden, aber ich glaube, dass man für seltene Erkrankungen auch eine solche Lösung schaffen kann und vielleicht sogar sollte. Und sich gerade auch im ambulanten Bereich auf vernünftige Finanzierungsfüße zu stellen.“*

Berücksichtigung der besonderen personellen und zeitlichen Aufwendungen	FG 2, Klinikverband	*„Wenn man sich von diesem Urteil mal ein bisschen frei macht und ein bisschen vielleicht Richtung 'was will denn die Politik eigentlich fördern?' und sich da ein bisschen öffnet, vielleicht kommt man dann eher in diese Vorstellung 'wir fördern auch den besonderen personellen Aufwand an Krankenhäusern, die das auch für ihre eigenen Patienten betreiben'."*
	FG 2, Patientenvertretung	*„Es geht meistens um sehr schwer erkrankte, komplex erkrankte Patienten, die einen hohen Aufwand an sprechender Medizin haben, also an Beratung, an Absprache, wie die weitere Therapie läuft, an Medikamenteneinstellung, die auch verbal begleitet werden muss, an Koordination des Teams, an Zusammenarbeit mit anderen Einrichtungen, also anderen Zentren, die dieselbe Indikation behandeln, auch an Zusammenarbeit mit nicht-ärztlichen Behandlern."*
	FG 1, B-Zentrum	*„Aber das ist nicht nur der Patient, der vor ihnen sitzt, der zeitlich aufwändig ist. Das ist das alles drum rum. Das Gerede mit zehn anderen Leuten, um herauszukriegen, wie man denen vielleicht mal helfen kann."*
Abbildung aufgewendeter Ressourcen als Verhandlungsgrundlage	FG 1, B-Zentrum	*„Vielleicht kriegt man diese Informationen leichter, wenn man sozusagen nicht fragt, wie viel Euro sind das, sondern, wenn man die Verwaltung anfragt, 'wie viel Zeit wird aufgewendet' oder 'was / wie groß ist so das Personal', ich weiß es nicht. Ich stelle mir vor, dass es vielleicht ein bisschen einfacher ist."*
	I 3, Kostenträger	*„Ich würde sagen, man muss die Leistung darstellen. Welche Leistung soll erfasst werden? Die Leistung muss man dann halt verhandeln. Ist sie erforderlich? Ist sie nicht erforderlich? In welchem Umfang, wie oft? Und dann muss man die Leistung nachher in Euro bewerten."*
Präferenz für pauschalierte Vergütung	FG 2, Klinikverband	*„Wo man natürlich auch sagen muss, das ist am Ende auch immer noch eine Pauschale, dass man da auch dem Einzelfall, auch beispielsweise der [Name Erkrankung]-Versorgung, nicht im Einzelfall gerecht wird, muss man auch klar sein, soweit werden wir nie kommen. Warum werden wir nie so weit kommen? Wir werden nicht das DRG-System auf die Hochschulambulanzen transferieren können gedanklich, weil das landet in einem bürokratischen Supergau, den keiner mehr administrieren kann. Also insofern haben Pauschalen natürlich auch unter der Abwägung zwischen Leistungsgerechtigkeit und bürokratischem Aufwand / muss man da auch den richtigen Weg für finden."*

Sonderpauschale für seltene Erkrankungen	FG 1, B-Zentrum	*„Und das ganz große Problem ist, im Prinzip brauchen sie eine Sonderpauschale oder sowas für seltene Erkrankungen im ambulanten Bereich. Die dann ein Uniklinikum aber auch ein nicht-universitäres Haus irgendwie on top kriegt, dass man sagt, für gewisse Krankheitsbilder, die eine Zentrumsstruktur haben, im B-Zentrum gibt es 200€ on top der Abrechnung, die sie sonst haben."*
	FG 1, B-Zentrum	*„Aber wir sind dabei, für die B-Zentren und für das Klinikum die Kosten zu kalkulieren sozusagen und dann zu überlegen, wie man dann eine besondere HSA-Pauschale verhandelt. Und da werden wir nicht für jedes B-Zentrum eine eigene haben, sondern werden versuchen, Gruppen zu bilden."*
Abgestufte Kalkulation der Sonderpauschale	I 3, Kostenträger	*„Man kann ja eine Pauschale machen, machen sie ja oft, über alle Fälle, wo dann halt gewichtet wird: leichte, mittlere und schwere Fälle. Das kann man ja dann von Jahr für Jahr auch sich immer wieder angucken und monitoren."*
